# Complement C7 and clusterin form a complex in circulation

**DOI:** 10.3389/fimmu.2024.1330095

**Published:** 2024-01-25

**Authors:** Mariam Massri, Erik J.M. Toonen, Bettina Sarg, Leopold Kremser, Marco Grasse, Verena Fleischer, Omar Torres-Quesada, Ludger Hengst, Mikkel-Ole Skjoedt, Rafael Bayarri-Olmos, Anne Rosbjerg, Peter Garred, Dorothea Orth-Höller, Zoltán Prohászka, Reinhard Würzner

**Affiliations:** ^1^ Institute of Hygiene & Medical Microbiology, Medical University of Innsbruck, Innsbruck, Austria; ^2^ R&D Department, Hycult Biotechnology, Uden, Netherlands; ^3^ Institute of Medical Biochemsitry, Protein Core Facility, Biocenter, Medical University of Innsbruck, Innsbruck, Austria; ^4^ Institute of Medical Biochemistry, Medical University of Innsbruck, Biocenter, Innsbruck, Austria; ^5^ Tyrolean Cancer Research Institute, Innsbruck, Austria; ^6^ Laboratory of Molecular Medicine, Department of Clinical Immunology, Rigshospitalet, Copenhagen University Hospital, Copenhagen, Denmark; ^7^ Institute of Immunology & Microbiology , University of Copenhagen, Copenhagen, Denmark; ^8^ MB-LAB Clinical Microbiology Laboratory, Innsbruck, Austria; ^9^ Department of Internal Medicine and Hematology, Semmelweis University, Budapest, Hungary; ^10^ Research Group for Immunology and Hematology, Semmelweis University-Eötvös Loránd Research Network (Office for Supported Research Groups), Budapest, Hungary

**Keywords:** complement C7, clusterin, complex, complement, circulation

## Abstract

**Introduction:**

The complement system is part of innate immunity and is comprised of an intricate network of proteins that are vital for host defense and host homeostasis. A distinct mechanism by which complement defends against invading pathogens is through the membrane attack complex (MAC), a lytic structure that forms on target surfaces. The MAC is made up of several complement components, and one indispensable component of the MAC is C7. The role of C7 in MAC assembly is well documented, however, inherent characteristics of C7 are yet to be investigated.

**Methods:**

To shed light on the molecular characteristics of C7, we examined the properties of serum-purified C7 acquired using polyclonal and novel monoclonal antibodies. The properties of serum‑purified C7 were investigated through a series of proteolytic analyses, encompassing Western blot and mass spectrometry. The nature of C7 protein-protein interactions were further examined by a novel enzyme-linked immunosorbent assay (ELISA), as well as size‑exclusion chromatography.

**Results:**

Protein analyses showcased an association between C7 and clusterin, an inhibitory complement regulator. The distinct association between C7 and clusterin was also demonstrated in serum-purified clusterin. Further assessment revealed that a complex between C7 and clusterin (C7-CLU) was detected. The C7-CLU complex was also identified in healthy serum and plasma donors, highlighting the presence of the complex in circulation.

**Discussion:**

Clusterin is known to dissociate the MAC structure by binding to polymerized C9, nevertheless, here we show clusterin binding to the native form of a terminal complement protein in vivo. The presented data reveal that C7 exhibits characteristics beyond that of MAC assembly, instigating further investigation of the effector role that the C7-CLU complex plays in the complement cascade.

## 1 Introduction

Complement is an intricate system that plays an important role in host homeostasis and host defence against invading pathogens (1). Complement activation initiates sequential cleavage of complement proteins, eliciting an inflammatory response that leads to pathogen killing. Complement is activated via three pathways: the classical, lectin and alternative pathways (2, 3). The pathways converge at the formation of the C3 convertase and the subsequent cleavage of C3 into the opsonizing component C3b and the anaphylatoxin C3a. All three pathways finally trigger the activation of the terminal complement pathway, characterized by the formation of a membrane attack complex [MAC; also known as terminal complement complex (TCC or C5b-9)] on target cell membranes (2). The MAC consists of an initiating C5b6-7 complex that tethers onto the cell surface, followed by the addition of C8 and 12-18 molecules of C9, inducing pore formation on or within a cell surface (4, 5). This results in pathogen elimination (6) or, if the MAC is present in lower numbers, sub-lytic activities (7).

Complement C7 is a glycoprotein composed of a single polypeptide chain with a molecular weight of 95-100 kDa (8). It consists of 9 protein domains, which are highly homologous between the terminal complement proteins C6, C7, C8 and C9 (9). C7 is one of the essential components involved in the assembly of the MAC. The binding of C7 to the C5b6 complex is responsible for the transition of the hydrophilic structure into an amphiphilic C5b7 complex, thereby enabling the complex to bind target membranes. C8 and polymerized C9 assemble onto the tethered C5b7 complex, resulting in the pore-forming and lytic MAC (8, 10).

Certain polymorphisms in the C7 gene, including base-pair deletions (11, 12), exon deletions (13, 14) and nonsense variants (15, 16), result in truncated variants which prevent the proper translation and secretion of C7 into circulation, leading to C7 deficiencies and dysregulations (17, 18), some of which lead to subtotal deficiencies (19). One potential variant of C7 that has not been thoroughly investigated was first discovered by one of our lab members (RW) 35 years ago. At that time, C7 was observed as a double band in Western blot analysis when native C7 was probed with anti-C7 antibodies; one band at around 100 kDa, representing the length of native C7, and another unknown band around 75 kDa (unpublished data). It was hypothesized that alternative splicing in the C7 gene explains the co-presence of a full and truncated form of C7. We previously reported on an extensive mapping of single nucleotide polymorphisms (SNPs) in the C7 gene, where we identified a particular SNP (rs74480769) that could potentially lead to a truncated C7 protein (18). The SNP is located in intron 14 of the C7 gene and corresponds to the last nucleotide (A/G) of an alternative transcript featuring a cassette exon. Bioinformatic predictions revealed that the rs74480769 SNP has the potential to induce an alternative splicing of the C7 gene, thereby introducing a premature stop codon in frame and the truncation of the C7 protein. Notably, given the position of the SNP, the resultant truncated C7 would be 75% of the total length of native C7. Taking these observations together, we hypothesized that the 75 kDa band produced by purified C7 pinpoints towards a truncated C7 that is 75% the length of the mature 100 kDa C7 protein. Furthermore, since a truncated C7 is incapable of incorporating within the MAC, as the C-terminal sequences of C7 are essential in MAC assembly (20), it was additionally hypothesized that the variant possesses regulatory functions.

This study aimed to assess the nature of the 75 kDa band by analysing C7 purified from serum. We similarly showcased two bands for purified C7 at around 100 kDa and 75 kDa. Nevertheless, we did not observe a variant form of C7, instead, our analysis of the 75 kDa band revealed an abundance of the complement regulator clusterin, and further investigation indicated the presence of a C7-clusterin (C7-CLU) complex in circulation. The novel protein complex characterized in this study highlights that C7, complexed with clusterin, could play a distinct role in circulation.

## 2 Materials and methods

### 2.1 Reagents

Antibodies: goat anti-human C7 polyclonal antibody [P7] (21); mouse anti-human C7 monoclonal antibody [M7-WU4-15] (cat# HM2277, Hycult Biotech, Uden, Netherlands); mouse anti-human C7 monoclonal antibody [M7-HB2H] (cat# HM2421, Hycult Biotech), goat anti-human clusterin polyclonal antibody [PC] (Sigma-Aldrich, St. Louis, MO, USA); mouse anti-human clusterin monoclonal antibody [MC] (Sinobiological, Beijing, China), mouse anti-human clusterin monoclonal antibody [MC-2D5] (cat# HM2435, Hycult Biotech); goat anti-mouse HRP-conjugated IgG antibody (Thermo Fisher Scientific, Waltham, MA, USA). Commercially-purified C7 and C7 depleted sera were from Complement Technology (Tyler, TX, USA). Recombinant clusterin was from R&D systems (Minneapolis, MN, USA).

### 2.2 Generation of the C7 monoclonal antibody M7-HB2H

**Figure 1:**
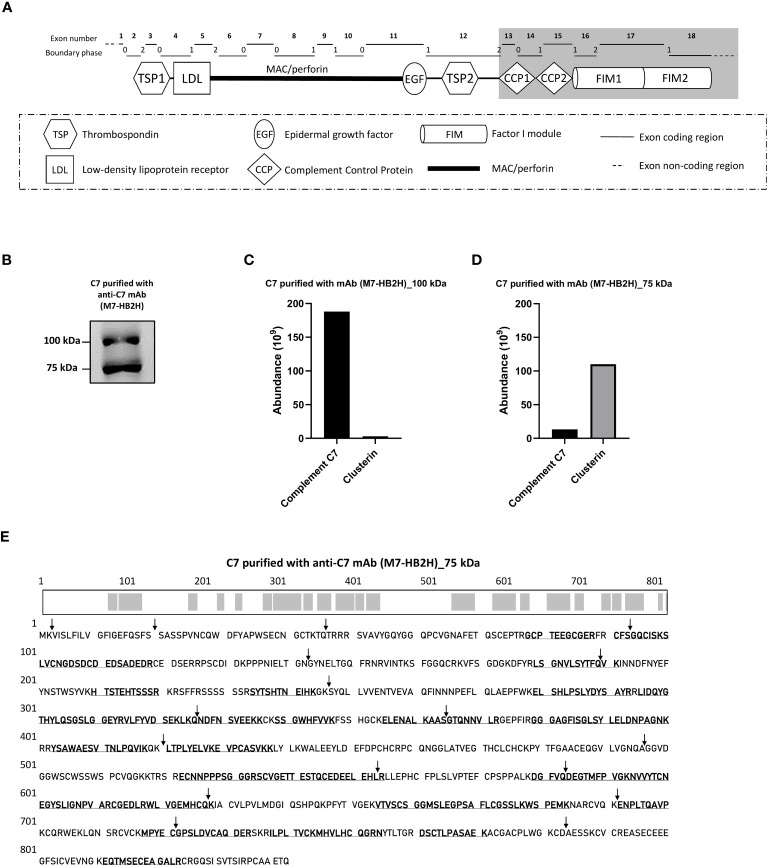
The C7 and clusterin association was confirmed with a native-restricted anti-C7 mAb. **(A)** The M7-HB2H mAb binding target is located in the C-terminal domains (highlighted in grey) of C7. These domains participate in the formation of the MAC via interaction with the C345C domain of C5, hence, the M7-HB2H antibody would likely bind to an exposed epitope in the native form of C7 (i.e. C7 that is not involved in the assembly of the MAC). The C7 gene contains 18 exons, each exon codes for a specific region of the C7 domain, which corresponds to the exon coding region displayed above. The boundary phase refers to the phase of the corresponding intron. **(B)** C7 purified from NHS-BioIVT with anti-C7 mAb, M7-HB2H, was separated by SDS-PAGE under non-reducing conditions and the immunoblot was probed with M7-HB2H. Protein composition of the bands visualized in **(B)** was analzyed by mass spectrometry and protein abundance was quantified for the **(C)** 100 kDa band and the **(D)** 75 kDa band. **(E)** The C7 aa residues detected in the 75 kDa band (3B) are in bold and underlined, along with an illustration of the screened residues in the C7 polypeptide chain highlighted in grey. The screened aa residues are 47% of the total C7 polypeptide chain. Black arrows indicate the exon/intron boundaries. Western blot **(B)** is representative of at least three independent experiments. Images were taken using Proxima C16 Phi. Figure **(A)** modified from Massri et al., 2022 (18).

The animal experimental procedures described in this study have been approved by the Danish Animal Experiments Inspectorate with the approval ID: 2019-15-0201-00090. Outbred NMRI mice (n = 8) were subcutaneously immunized with a peptide comprised of the C-terminal domains of C7 (**Figure 1A**). The mice received two doses of the antigen at a concentration of 1µg/mL with a 2-week interval between each dose. Splenocytes collected from immunized mice underwent a fusion and selection process following the principles of hybridoma technology (22), as previously described (23). Positive clones were screened in MaxiSorp microtiter plates (Thermo Fisher Scientific) coated with 1μg/mL of the C7 antigen followed by an HRP-conjugated rabbit anti-mouse IgG (1:2000; Dako, Santa Clara, CA, USA). Positive clones were sent to Hycult Biotech for hybridoma screening and testing for antibody specificity. Briefly, hybridoma clones were cultured in 24-well cell culture plates for 7 days at 37°C and 5% CO_2_. Clones that displayed positive growth were identified by measuring the concentration of IgG production using IgG-coated ELISA plates. Following a second round of culturing for 7 days, positive clones that displayed 100% IgG production were subsequently tested for antibody specificity via direct antigen ELISA (i.e. plates coated with the C7 peptide). Positively selected clones underwent a scale-up production, and ultimately, monoclonal antibodies were purified using protein G columns.

### 2.3 Collection and preparation of pooled normal human serum

As many other proteins, C7 polymorphisms may be present in circulation, and one of the most known is the C7 M/N polymorphism (24). The C7M allotype is defined by a threonine residue, while C7N is defined by the relatively smaller proline residue at aa 587. Hence, the binding properties of C7 may be influenced by the type of C7 allotype present. In order to encompass these known C7 variants, pooled NHS was used instead of individual serum donors. Experiments were performed using three independent sources of pooled normal human serum (NHS). Serum was acquired from fresh blood of healthy human donors, with informed consent, under the approval of the Ethics Committee at the Medical University of Innsbruck, Austria (ECS1166/2018, 14 November 2018). Blood from at least 6-8 healthy donors was collected and left for 30 minutes at room temperature to allow the blood to clot, before centrifuging it at 1377 xg for 15 minutes. Supernatant from individual donors was pooled and centrifuged once more to acquire the final designated serum [NHS-MUI]. Purification of C7 and clusterin with M7-HB2H and MC-2D5, respectively, was performed using NHS purchased from BioIVT [NHS-BioIVT] (Westbury, NY, USA). NHS pool prepared at Semmelweis University, under the approval of the Scientific and Research Ethics Committee of the Medical Research Council (8361-1/2011-EKU) in Budapest, Hungary, was used for experiments performed with activated NHS. Zymosan-activated NHS was prepared by incubating NHS with *Saccharomyces cerevisiae* (Thermo Fisher Scientific) at 37°C and the reaction was subsequently stopped by 25mM of ethylenediaminetetraacetic acid (EDTA).

### 2.4 Protein purification by co-immunoprecipitation

C7 and clusterin were purified from NHS using the Thermo Scientific Pierce™ Co-Immunoprecipitation (Co-IP) Kit. C7 was purified using either the anti-C7 polyclonal antibody (pAb) [P7], the anti-C7 monoclonal antibody (mAb), M7-WU4-15, or the anti-C7 mAb, M7-HB2H. The antibody was covalently coupled to AminoLink Plus Coupling Resin in a Pierce™ Spin Column. A total of 500µl of NHS was treated with Pierce™ Control Agarose Resin for 1 hour at 4°C, to reduce nonspecific protein binding before it was incubated with the antibody-coupled resin spin column. After an overnight incubation at 4°C, the spin column was centrifuged to collect the NHS and subsequently washed with the Pierce™ IP Lysis/Wash Buffer according to the kit manual. The antibody-bound C7 was immunoprecipitated using the Pierce™ Elution Buffer and stored at 4°C. The Co-IP method was performed two more times, using new NHS each time. Clusterin was purified with the anti-clusterin pAb, PC, or the anti-clusterin mAb, MC-2D5, using the same Co-IP technique described for the purification of C7. The flow-through of NHS acquired, after capturing clusterin in the column, served as our clusterin depleted sera. The specificity of Co-IP columns in purifying C7 and clusterin was validated by evaluating the eluate from an IgG isotype antibody (**Supplementary Figure 2**).

### 2.5 Concentration and dialyzation of purified proteins

Purified C7 and clusterin samples were concentrated and dialyzed using Vivaspin^®^ 500µl ultrafiltration spin columns (Sartorius, Göttingen, Germany) with an exclusion pore of 30 kDa. The purified proteins were concentrated 6x followed by dialysis with Milli-Q water. A final 10x concentration step was performed and the purified proteins were stored at -20°C until further analysis.

### 2.6 Gel electrophoresis and Western blot

Purified proteins were separated by sodium dodecyl sulphate-polyacrylamide gel electrophoresis (SDS-PAGE) using Novex™ WedgeWell™ 4 to 20%, Tris-Glycine, 1.0 mm, Mini Protein Gels (Invitrogen, Waltham, MA, USA). Non-reduced samples were diluted in Laemmli Sample buffer (Sigma-Aldrich). Reduced samples were diluted in Laemmli Sample Buffer with 200mM dithiothreitol and incubated at 95°C for 5 minutes. Gel electrophoresis was followed by Western blot transfer to polyvinylidene fluoride membrane (Bio-rad, Hercules, CA, USA). The membranes were probed with anti-C7 or anti-clusterin antibodies, as indicated in the figure legends, and detected with a goat anti-mouse HRP-conjugated IgG antibody. Membranes were visualized with enhanced chemiluminescence (ECL) substrate (Bio-Rad). Images were taken with the ImageQuant™ Las 4000 camera system (GE Healthcare, Chicago, IL, USA) or Proxima C16 Phi (Isogen Life Sciences, Utrecht, Netherlands).

### 2.7 Protein sequencing by liquid chromatography-mass spectrometry

Purified proteins were separated by SDS-PAGE under non-reducing conditions and protein bands were visualized by Coomassie Brilliant Blue staining (Sigma-Aldrich). Coomassie-stained bands were excised from the SDS-PAGE gel, reduced with dithiothreitol, alkylated with iodoacetamide and digested with trypsin as previously described (25). Tryptic digests were analyzed using an UltiMate 3000 RSCLnano-HPLC system coupled to a Q Exactive HF mass spectrometer (both from Thermo Fisher Scientific, Bremen, Germany) equipped with a Nanospray Flex ionization source as previously described (26).

Purified proteins that were analyzed directly, without separation by SDS-PAGE, were analyzed as described above with the exception that the UltiMate 3000 RSCLnano-HPLC system was coupled to an Orbitrap Eclipse mass spectrometer (Thermo Fisher Scientific, Bremen, Germany). The Orbitrap Eclipse mass spectrometer equipped with a field asymmetric ion mobility spectrometer interface was operating in the data dependent mode with compensation voltages of -45 and -65 and a cycle time of one second. Survey full scan MS spectra were acquired from 375 to 1500 m/z at a resolution of 240,000 with an isolation window of 1.2 mass-to-charge ratio (m/z), a maximum injection time (IT) of 50 ms, and an AGC target of 400,000. The MS2 spectra were measured in the Orbitrap analyzer at a resolution of 15,000 with a maximum IT of 22 ms and an AGC target of 50,000. The selected isotope patterns were fragmented by higher-energy collisional dissociation with normalized collision energy of 28.

### 2.8 Analysis of C7-CLU complex by ELISA

The C7-CLU complex was assessed using a custom sandwich enzyme-linked immunosorbent assay (ELISA), which will be under development by Hycult Biotech in the future. Nunc MaxiSorp ELISA plates (Thermo Fisher Scientific) were coated with a monoclonal antibody against the C7 protein and blocked with 1% bovine serum albumin. Samples were incubated for 30 minutes at room temperature and bound C7-CLU complex was detected with an HRP-conjugated monoclonal antibody against clusterin. The assay was developed using 3, 3’, 5, 5’-tetramethylbenzidine (TMB) substrate and the absorbance, defined by optical density (OD), was measured at 450 nm using a spectrophotometer. Serum-purified C7 was used as a standard for the measurement of the C7-CLU complex, and defined by arbitrary units (AU)/mL, where 1 AU/mL is approximately 1 ng/mL. The C7-CLU complex was measured in a 4-point serial dilution. The linearity of the dilutions was monitored by calculating the coefficient of variation (%CV), where %CV < 20% was considered a low variation.

### 2.9 Purification of the C7-CLU complex by size-exclusion chromatography

Pooled NHS was fractionated by gel filtration chromatography. First, NHS was ultracentrifuged at 50.000 rpm for 2 h at 4°C (Optima TLX ultracentrifuge, Beckman, Coulter, Baierbrunn, Germany) to extract the lipoproteins and to precipitate large complexes and aggregates. Samples were then injected in an ÄKTA Prime Plus purification system (Cytiva, Freiburg, Germany), previously equilibrated in PBS (150 mM NaCl, pH 7.4) and separated on a Superdex 200 HR 10/30 column (546126, Cytiva). Fractions (0.3 ml) were collected with a flow rate of 0.1 ml/min at 4°C by isocratic elution over 1.0 column bed volume. Gel filtration molecular markers (MWGF200, Sigma-Aldrich) were fractionated in the same manner as the samples using the 200 kDa (β-amylase), 150 kDa (yeast alcohol dehydrogenase) and 66 kDa (bovine serum albumin) size markers. Fractions were subsequently analyzed by Western blot and ELISA. Data was analyzed using the PrimeView software (Cytiva).

### 2.10 Statistical analysis

All statistical analyses and data plotting was performed on GraphPad Prism version 9.4.0 (San Diego, CA, USA). Comparison between multiple groups was conducted using one-way analysis of variance (ANOVA) followed by Tukey’s test to correct for multiple comparisons. Dunnett’s *post-hoc* test was used for the comparison of zymosan-activated NHS against the non-activated control. *P* values < 0.05 were considered statistically significant. Results are presented as mean ± standard deviation (SD).

## 3 Results

### 3.1 Detection of a variant of C7 by immunoblotting

**Figure 2:**
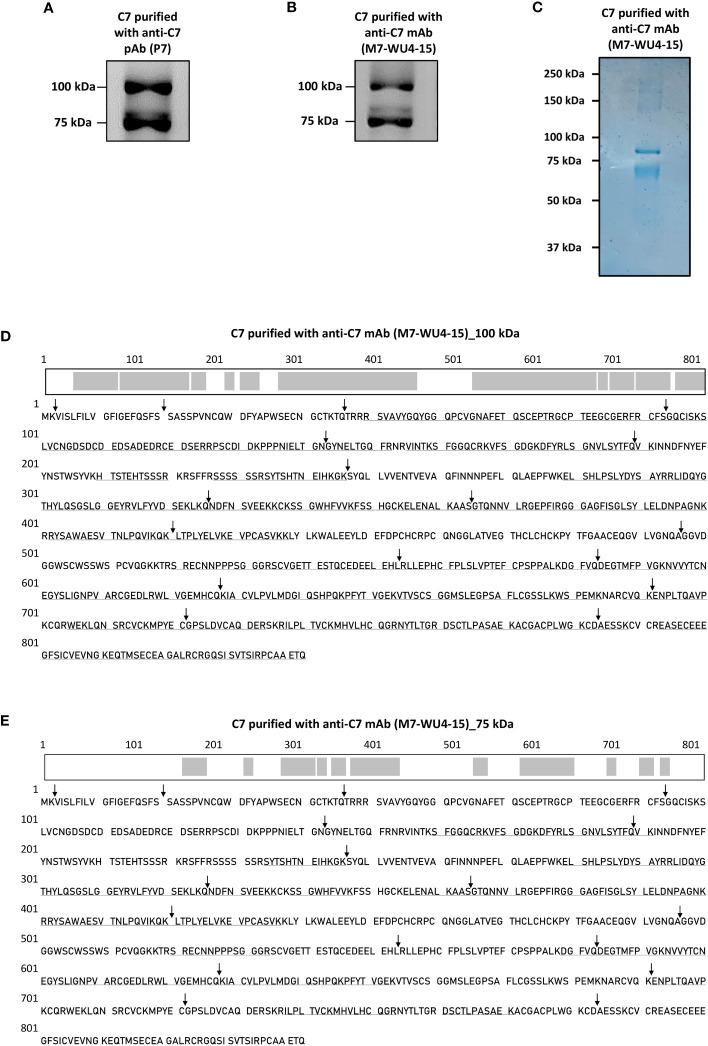
Detection of a variant form of C7 and screening of C7 aa residues in the visualized variant. **(A)** C7 purified from NHS-MUI with the anti-C7 pAb, P7, was separated by SDS-PAGE under non-reducing conditions and immunoblot was probed with the anti-C7 mAb, M7-WU4-15. **(B)** C7 purified from NHS-MUI with M7-WU4-15 was separated by SDS-PAGE under non-reducing conditions and immunoblot was probed with M7-WU4-15. **(C)** Coomassie staining of C7 purified from NHS-MUI with the anti-C7 mAb, M7-WU4-15, and separated by SDS-PAGE gel under non-reducing conditions. **(D)** The C7 aa residues identified by LC-MS in the 100 kDa band (1B) are in bold and underlined, along with an illustration of the screened residues in the C7 polypeptide chain highlighted in grey. The screened aa residues are 76% of the total C7 polypeptide chain. **(E)** The C7 aa residues detected in the 75 kDa band (1B) are in bold and underlined, along with an illustration of the screened residues in the C7 polypeptide chain highlighted in grey. The screened aa residues are 37% of the total C7 polypeptide chain. Black arrows indicate the exon/intron boundaries. Western blots and coomassie staining are representative of at least three independent experiments. Images were taken using the ImageQuant™ LAS 400 camera system.

To identify the composition of the 75 kDa of C7, described previously by RW, C7 was purified from NHS-MUI using the anti-C7 pAb, P7 and applied to SDS-PAGE under non-reducing conditions. The immunoblot was probed with the anti-C7 mAb, M7-WU4-15. We also observed two bands for the purified C7, one at around 100 kDa and another at 75 kDa (**Figure 2A**). To ensure that our results were not caused by unspecific proteins co-purified with C7, we repeated the purification using the anti-C7 mAb M7-WU4-15, which recognizes the C7M allotype; an allotype characterized by the presence of threonine at amino acid (aa) position 565 (updated aa position is 587) (18) due to a missense substitution (21). Bands at 100 kDa and 75 kDa were again observed when C7 was purified and probed with M7-WU4-15 (**Figure 2B**).

Both bands visualized by Coomassie staining (**Figure 2C**) were excised, digested and analysed by LC-MS. The resultant sequence coverage was 76% (**Figure 2D**) and 37% (**Figure 2E**) for the 100 kDa and 75 kDa bands, respectively. Given that C-terminal aa residues were detected (**Figure 2E**), it is unlikely that the 75 kDa band point towards a truncated C7 variant.

### 3.2 Clusterin is the main protein detected in the 75 kDa band of purified C7

**Figure 3:**
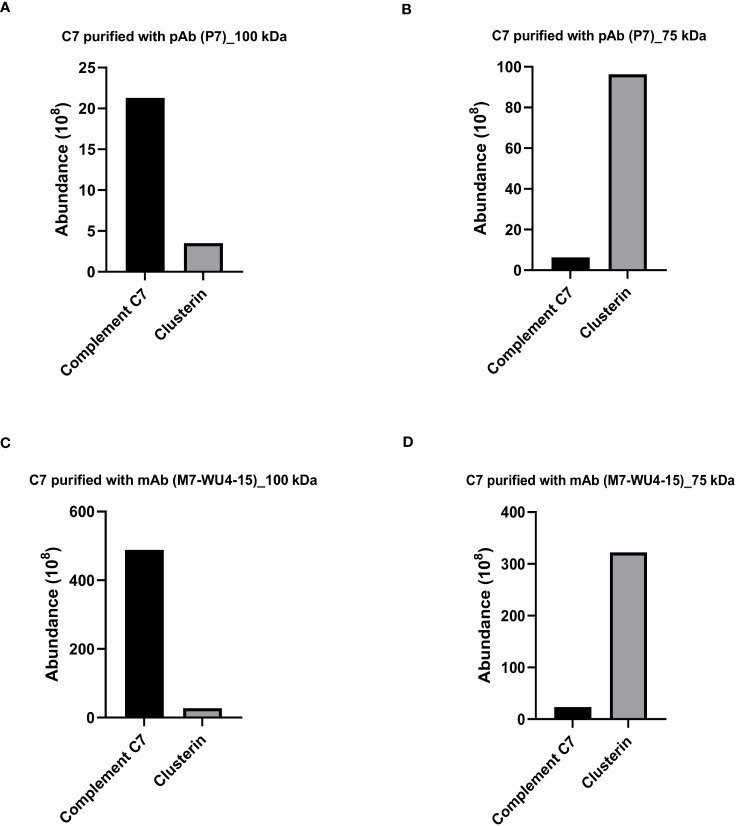
Clusterin is the most abundant protein detected in the 75 kDa band of purified C7. Protein composition of the bands visualized for C7 purified with pAb P7 (**Figure 2A**) was analzyed by mass spectrometry and protein abundance was quantified for the **(A)** 100 kDa band and the **(B)** 75 kDa band. Protein composition of the bands visualized for C7 purified with mAb M7-WU4-15 (**Figure 2B**) was analyzed by mass spectrometry and protein abundance was quantified in the **(C)** 100 kDa band and the **(D)** 75 kDa band.

To investigate the protein composition of the visualized bands at 100 kDa and 75 kDa, the bands were stained with Coomassie brilliant blue, excised and subsequently analyzed by LC-MS. The protein abundance was quantified by calculating the sum of all peptide ions for the detected proteins. For C7 purified and probed with the anti-C7 pAb, P7, the most abundant protein identified in the 100 kDa band was C7, while clusterin was the second most abundant protein (**Figure 3A**). In contrast, the 75 kDa band was most abundant in clusterin, while C7 was present in smaller concentrations (**Figure 3B**). Similar results were obtained for the 100 kDa and 75 kDa bands of C7 purified and probed with M7-WU4-15 (**Figures 3C, D**). However, the abundance of C7 and clusterin was higher in C7 purified with M7-WU4-15, compared to C7 purification with P7. Overall, our data do not point towards the presence of an alternatively spliced variant of C7 in the 75 kDa band as initially theorized. Instead, an association between C7 and clusterin was identified, and was investigated further.

### 3.3 Association between C7 and clusterin revealed with native-restricted C7 antibody

To further confirm the association between C7 and clusterin, C7 was purified from an independent NHS pool (NHS-BioIVT) using the newly-generated antibody, M7-HB2H, a native-restricted antibody that binds to the C-terminal domains of C7 (CCP1-FIM2) (**Figure 1A**). When purified C7 was applied to SDS-PAGE under non-reducing conditions and probed with M7-HB2H, we again observed two bands at 100 kDa and 75 kDa (**Figure 1B**). The most abundant protein observed in the 100 kDa was C7, followed by clusterin (**Figure 1C**). The reverse was observed in the 75 kDa band (**Figure 1D**).

We have repeatedly observed that the anti-C7 antibodies strongly detect the 75 kDa band, although it was confirmed that the band contains mostly clusterin. To investigate this, the 75 kDa band (observed in **Figure 1B**) was screened for the presence of C7 aa residues by mass spectrometry. The C7 sequence coverage in the band was 47% (**Figure 1E**), explaining the presence of the 75 kDa band when probed with the anti-C7 mAb, M7-HB2H. Notably, no specific bands were detected when the anti-C7 antibodies, M7-HB2H and M7-WU4-15, were blotted against C7 depleted serum (data not shown), verifying the specificity of the antibodies in recognizing the native C7 protein.

### 3.4 Low levels of C7 detected in clusterin purified with polyclonal and monoclonal antibodies

**Figure 4:**
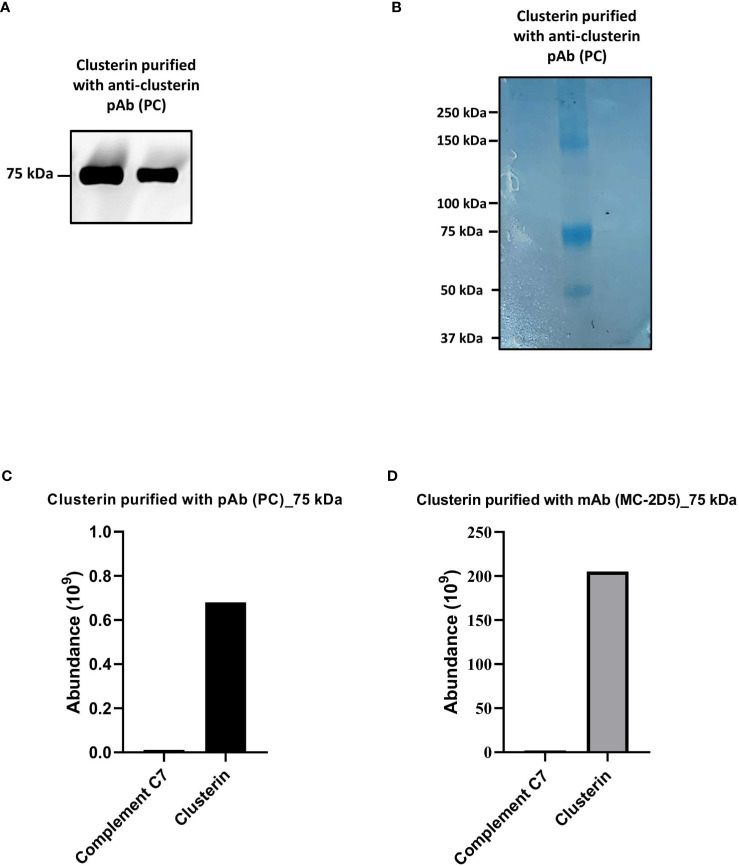
Clusterin purified from NHS with polyclonal and monoclonal antibodies contained low levels of C7. **(A)** Clusterin purified from NHS-MUI with anti-clusterin pAb, PC, was separated by SDS-PAGE under non-reducing conditions and the immunoblot was probed with the anti-clusterin mAb, MC. The clusterin band on the left was from the 1^st^ eluate acquired from the antibody-coupled column, while the band on the right was from a 2^nd^, consecutive elution step. **(B)** Coomassie staining of clusterin purified from NHS-MUI with the anti-clusterin pAb, PC, and separated by SDS-PAGE gel under non-reducing conditions. **(C)** Protein composition of the 75 kDa band visualized in **(A)** was analyzed by mass spectrometry and protein abundance was quantified. **(D)** Protein compostion of the 75 kDa band (not shown) from C7 purified with the anti-clusterin mAb, MC-2D5, was analyzed by mass spectrometry and protein abundance was quantified. Results from purified clusterin in **(B, C)** were acquiered from the 1^st^ eluate. Western blot and coomassie staining are representative of at least three independent experiments. Images were taken using the ImageQuant™ LAS 400 camera system.

We further investigated whether clusterin purified from NHS exhibits an association with C7. To assess this, clusterin was purified from NHS with the anti-clusterin pAb, PC, applied to an SDS-PAGE under non-reducing conditions and probed with the anti-clusterin mAb, MC. Western blot analysis revealed a prominent band at 75 kDa (**Figure 4A**). The 75 kDa band was further visualized by Coomassie staining (**Figure 4B**) and the band was excised, digested and analyzed by LC-MS. The most abundant protein detected in the 75 kDa band was clusterin, and although C7 was the second most abundant protein identified in the band, it was found at considerably low levels (**Figure 4C**). The band visualized around the 150 kDa mark (**Figure 4B**) comprised mostly IgG (data not shown).

To exclude the possibility that C7 was detected in purified clusterin due to potential cross-reaction with a pAb (i.e. PC), we measured the total protein abundance of clusterin purified with the mAb, MC-2D5. The protein profile revealed clusterin to be the most abundant protein, and although C7 was also detected, it was present at relatively low levels (**Figure 4D**).

### 3.5 C7 and clusterin association confirmed by Western blot analysis

**Figure 5:**
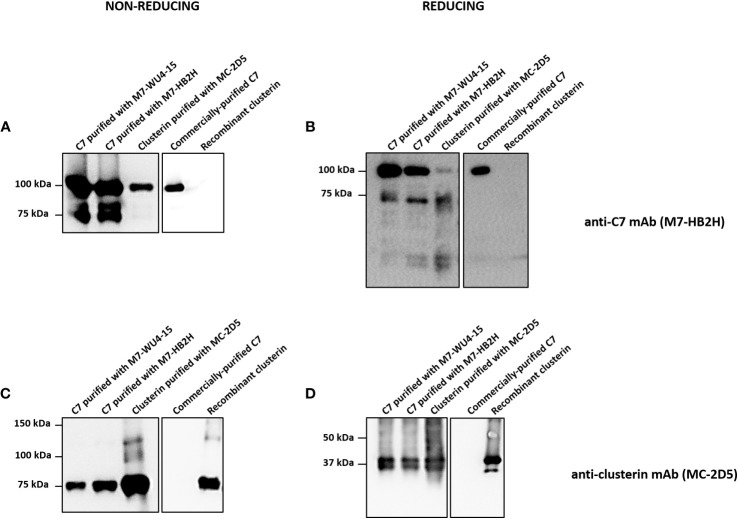
Western blot analysis reveals strong association between C7 and clusterin. **(A, B)** Indicated proteins were separated by SDS-PAGE under **(A)** non-reducing or **(B)** reducing conditions and immunoblots were probed with the anti-C7 mAb, M7-HB2H. **(C, D)** Indicated proteins were separated by SDS-PAGE under **(C)** non-reducing or **(D)** reducing conditions and immunoblots were probed with the anti-clusterin mAb, MC-2D5. Western blots are representative of at least three independent experiments. Images were taken using Proxima C16 Phi.

A series of Western blot experiments were performed to validate the mass spectrometry results, which demonstrated a definite association between C7 and clusterin. C7 purified with anti-C7 mAbs, M7-WU4-15 and M7-HB2H, and clusterin purified with the anti-clusterin mAb, MC-2D5, were analyzed in SDS-PAGE under non-reducing and reducing conditions. Commercially-purified C7 and recombinant clusterin served as controls. Upon several chromatographic steps during purification, no clusterin peptides were identified in the commercially-purified C7 upon mass spectrometry analysis (data not shown). When probed with M7-HB2H under non-reducing conditions, purified C7 samples produced bands at 100 kDa and 75 kDa (**Figure 5A**). Clusterin purified with MC-2D5 produced a band at 100 kDa when probed with M7-HB2H (**Figure 5A**), indicating the presence of C7 in purified clusterin. Under reducing conditions, C7 purified samples produced bands at 100 kDa and 75 kDa (**Figure 5B**). Given that C7 is a single polypeptide chain, it remains at 100 kDa even under reducing conditions (**Figure 5B**). Although purified clusterin produced a band at 100 kDa under non-reducing conditions, under reducing conditions we observed a more prominent band at 75 kDa and two bands at around 40 kDa (**Figure 5B**).

Purified C7 and clusterin were probed with the anti-clusterin mAb, MC-2D5, under non-reducing and reducing conditions (**Figures 5C, D**). Under non-reducing conditions, purified C7 samples produced a band at 75 kDa (**Figure 5C**), emphasizing the presence of clusterin in purified C7. Purified clusterin produced a band at 75 kDa when probed with MC-2D5 (**Figure 5C**). Under reducing conditions, all purified samples produced two bands at around 40 kDa (**Figure 5D**). This band pattern is a characteristic observed in clusterin probed under reducing conditions, as clusterin separates into its two subunits, alpha and beta, each at around 40 kDa (27). Although commercially-purified C7 was purified from serum, it does not contain any clusterin sequences, which was further confirmed by mass spectrometry (data not shown). Thus, no specific bands were observed when commercially-purified C7 was probed with the anti-clusterin antibody MC-2D5. This is due to the fact that commercially-purified C7 (prepared by Complement Technology) underwent several rounds of chromatographic steps. These results collectively demonstrate the robustness of the association between C7 and clusterin.

### 3.6 Custom ELISA demonstrates that C7 and clusterin form a protein complex

**Figure 6:**
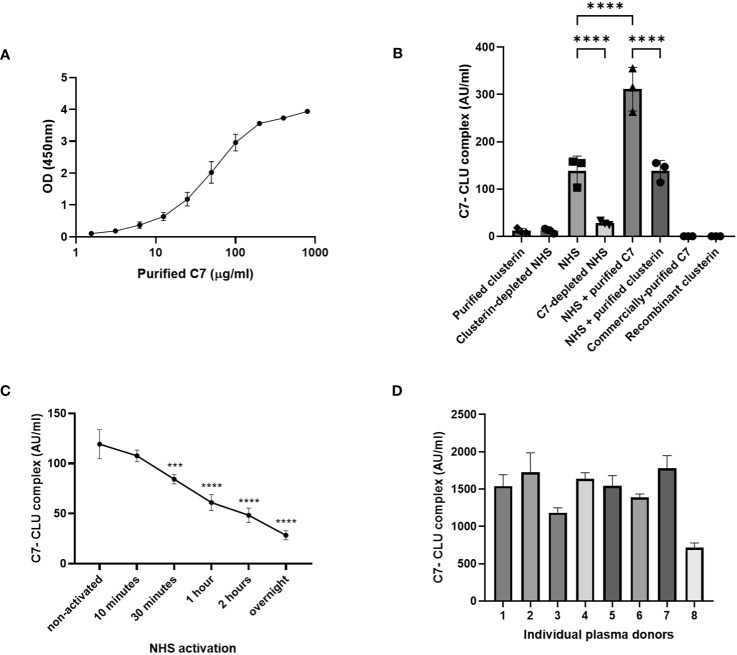
C7-CLU complex was detected in purified C7 and measured in healthy serum and plasma donors. **(A)** OD signal of C7-CLU complex measured in C7 purified from NHS-BioIVT with the anti-C7 mAb, M7-HB2H, at the indicated concentrations. **(B)** C7-CLU complex measured in indicated samples. Each data point denotes an independent measurement. **(C)** Concentration of C7-CLU complex measured zymosan-activated NHS at indicated time points. Asterisks denote statistically significant change from non-activated NHS. **(D)** Concentration of C7-CLU complex in EDTA plasma from healthy donors. Data are shown as mean ± SD. The CV values in all presented datasets were below 10%. Groups were compared using one-way ANOVA followed by Tukey’s multiple comparisons test. One-way ANOVA, followed by Dunnett’s multiple comparison test was used when comparing zymosan-activated NHS against non-activated control. *** *p* < 0.001, **** *p* < 0.0001. Some error bars cannot be shown because the SD is too small.

We demonstrated that C7 and clusterin exhibit an association, nevertheless, whether this association indicates that a direct complex is formed between the two proteins remains to be investigated. To this end, we established a custom sandwich ELISA to determine whether C7 and clusterin exist in a complex (C7-CLU). In brief, C7 was captured by an anti-C7 mAb, and clusterin, when bound to the captured C7, was detected with a HRP-labeled anti-clusterin mAb. Using this setup, we were able to detect the C7-CLU complex in serum-purified C7, and the signal intensity for the complex strongly correlated with the concentration of purified C7 (**Figure 6A**). Similar results were observed for C7 purified with M7-WU4-15 (data not shown).

Concentrations of the C7-CLU complex were measurably low in purified clusterin, and were comparable with clusterin-depleted NHS (**Figure 6B**). No signals were observed for both commercially-purified C7 and recombinant clusterin (**Figure 6B**).

### 3.7 Quantification of the C7-CLU complex in healthy serum and plasma donors

Next, our custom sandwich ELISA was used to measure the C7-CLU complex in both NHS and plasma samples acquired from healthy volunteers. We were able to measure the C7-CLU complex directly in serum from healthy donors. Levels of C7-CLU complex were elevated in NHS, compared to C7-depleted NHS (**Figure 6B**). Incubating NHS with purified C7 resulted in a marked increase in C7-CLU levels, suggesting that the purified complex was not degraded by any components in NHS (**Figure 6B**). C7-CLU levels in NHS incubated with purified clusterin were comparable with NHS (**Figure 6B**). On the other hand, activation of NHS with zymosan resulted in an overall decrease of the concentration of the complex, compared to non-activated NHS (**Figure 6C**). Notably, the concentration of the C7-CLU complex decreased in a time-dependent manner, indicating that complement activation reduces the detection of the complex.

Lastly, we assessed whether we could detect the complex not just in serum, but also in a sample preparation of EDTA-plasma. We measured the C7-CLU complex in a small subset of 8 EDTA-plasma samples obtained from healthy donors. Results show that the concentration of the complex ranged between 700 and 1800 AU/ml (**Figure 6D**).

### 3.8 The C7-CLU complex co-elutes at a higher molecular weight than expected

**Figure 7:**
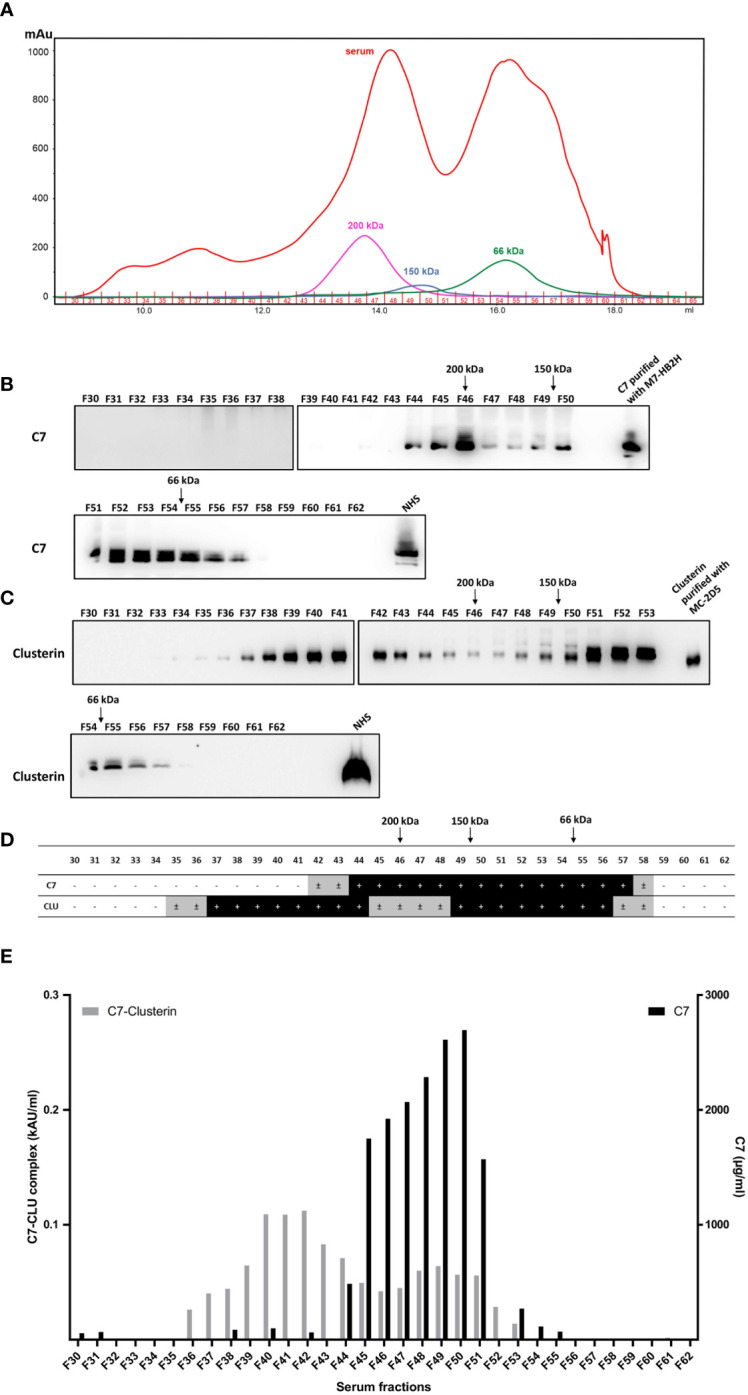
C7 and CLU co-elute in high molecular weight complexes. **(A)** Fractions of pooled NHS (F30-F62) were obtained by size exclusion chromatography and subsequently separated by SDS-PAGE under non-reducing conditions and immunoblots were probed with **(B)** anti-C7 mAb, M7-HB2H or **(C)** anti-clusterin mAb, MC-2D5. **(D)** Summary of **(B, C)** illustrating the fractions that were either positive or negative for C7 and clusterin. ± indicates fractions where a very faint band was detected due to minute amounts of the protein. **(E)** Concentration of the C7-CLU complex and native C7 in serum fractions. Note the difference between the y-axis scales, which translates to small concentrations of complexed C7, relative to native C7. NHS, normal human serum.

Thus far, the C7-CLU complex was characterized via the capture of the complex using monoclonal antibodies. To further confirm complex formation between C7 and clusterin, the C7-CLU complex was isolated based solely on its molecular weight. To this end, size exclusion chromatography was used to fractionate pooled NHS, where 32 fractions were collected (**Figure 7A**) and subsequently analyzed by Western blot and ELISA. Importantly, quantification of the exact size of proteins with high molecular weights (e.g., 200 kDa peak) is generally broad and extended to 8 fractions (2.4 ml). Furthermore, protein structure and association, within the column, may change its separation during SEC, which makes it difficult to determine the molecular weight precisely.

No C7 or clusterin was detected in fractions between F30-F34. Taking the aforementioned limitations into account, C7 was detected within a molecular range between 50-350 kDa, and the majority of C7 protein was detected at as size range between 100-200 kDa (**Figure 7B**). Clusterin was detected in the majority of fractions ranging between 66 kDa and 350 kDa (**Figure 7C**). A comparison between fractions containing C7 and clusterin is illustrated in **Figure 7D**. The peaks of native C7 detected in the immunoblot were further corroborated by measuring the concentration of C7 in each fraction (**Figure 7E**). Concentrations of the C7-CLU complex peaked in fractions that, according to the marker proteins, have a much higher molecular weight than expected (i.e. above 200 kDa), indicating that the complex is not composed of one molecule of C7 and clusterin each. It is important to stress that only around 6% of C7 is found complexed to clusterin, relative to the concentration of native C7 in serum fractions. Furthermore, the fact that these fractions contained little to no native C7 (i.e. F40-F42; Figure E) was expected, as native C7 peaks around 100 kDa and the amount of the C7-CLU complex was very low, compared to native C7, explaining the absence of C7 in these fractions in Western blots (**Figure 7B**). In contrast, the non-reducing – albeit denaturing – conditions of the SDS-PAGE produced a peak for clusterin at a very higher molecular weight, indicating that considerable amounts of clusterin are either complexed with another clusterin molecule, or different proteins, which could withstand denaturing conditions. In contrast, the clusterin peaks at the lower molecular weight would correspond with that of native clusterin.

## 4 Discussion

To date, there are a few studies that focus on dissecting the properties of C7 beyond its integral role in MAC assembly. In order to better understand the role of C7, we investigated the molecular characteristics of C7 purified from human serum. Our Western blot analysis of serum-purified C7 revealed an additional form of C7 with a molecular weight (75 kDa) lower than that of native C7 (100 kDa). Initially, we believed that a truncated C7 variant, due to alternative splicing, existed in circulation. However, given that we detected C-terminal residues in the 75 kDa band, we excluded the presence of a functionally truncated variant, as truncated proteins do not comprise end terminal sequences. Further analysis of the 75 kDa band by mass spectrometry revealed an abundance of clusterin, and a strong association between C7 and clusterin was identified.

Clusterin (also known as apolipoprotein J) is a ubiquitously expressed apolipoprotein, with various paradoxical functions (28). Several isoforms are generated from the clusterin protein precursor, the largest being a glycosylated form of α-β heterodimers (75-80 kDa) that is secreted from cells (29, 30). Clusterin is the first molecule identified to possess chaperone activities outside the cell (31). It is involved in the clearance of misfolded proteins from the extracellular matrix by binding to pathogenic protein aggregates, inducing their internalization and degradation (32, 33).

Clusterin inhibits the terminal complement pathway by binding C5b-9, resulting in a lytically inactive complex (34). C7, C8β and the b domain of C9 possess high affinity binding sites for clusterin, hence, the binding site is conserved between terminal complement proteins (35). Early descriptions state that clusterin inhibits C5b-6 initiated hemolysis (36), as well as complement-lysis activity via binding to nascent C5b-7 before it attaches onto a target membrane (37, 38). However, ligand blot analysis revealed that polymerized C9 competes for clusterin binding, thereby inhibiting clusterin interaction with other complement proteins. Consequently, clusterin was shown to inhibit TCC by binding to exposed sites on polymerized C9, and not to circulating native terminal complement proteins (35, 39). The presence of a neo-epitope on C9, exposed only upon formation of nascent C5b-9 or membrane-bound C5b-9 (40, 41), further supports the competitive binding of clusterin to exposed sites on polymerized C9. Overall, these studies do not suggest that clusterin binds circulating terminal complement proteins.

Here, we provided evidence of a complex between clusterin and a terminal complement protein, C7, in circulation. The complex between C7 and clusterin was observed using polyclonal, allotype-specific and native-restricted C7 antibodies across three independent pools of serum donors. This process limited the possibility of detecting artifact protein-protein interactions due to cross-reactive antibodies. Furthermore, using mAbs that only bind epitopes on native C7 ensured that C5b-7 components, or any of the intermediary components leading to the formation of the MAC (42, 43), were not co-purified. This was further corroborated by mass spectrometry analysis, as C7 was the only terminal complement protein detected with significant coverage (**Supplementary Table 1**). Thus, we ruled out the possibility that clusterin co-purification was derived from C5b-9-bound clusterin. However, whether clusterin forms a complex with other terminal complement proteins must be further investigated, given that C7, C8 and C9 possess a binding site to clusterin, as mentioned above. We are currently investigating the potential association of native C9 with clusterin; however, preliminary experiments do not suggest that purified C9 forms a complex with clusterin (**Supplementary Figure 3**).

We further performed experiments using multiple NHS pools in order to expand the profile of individuals with varying C7 polymorphisms. The C7 gene contains numerous single nucleotide variants, some of which are rare within a given population (18). The mapping and characterization of C7 variants unveiled several abnormalities in the C7 protein, including variants that could alter the binding properties of C7 (44). By the same token, we considered that clusterin association with C7 may be an exceptional phenomenon caused by a genetic variant, giving rise to a C7 protein that harbors uncharacteristic binding properties. However, the interaction between C7 and clusterin, and the measurement of the C7-CLU complex, was confirmed in independent NHS pools as well as in individual plasma donors. We hence concluded that the association between these two proteins was not due to C7 polymorphism, but that it occurs within a normal population.

Size exclusion chromatography is a standard method used to characterize protein complexes in medical fluids (45, 46), without the need to manipulate the environment, such as in the case of capture antibodies. SEC analysis therefore was used to validate the presence of the C7-CLU complex in circulation, revealing that the molecular weight of the complex was much higher than the expected range of 200 kDa. It is important to highlight that the majority of circulatory C7 is not bound to clusterin, as the concentration of the C7-CLU complex was a fraction of serum concentrations of native C7. Looking at the size, the complex may be slightly bigger than a complex composed of one molecule of C7 and clusterin each. Dimerization of complement molecules, such as CFHR proteins (47), is not a novel phenomenon. A dimer macromolecule of the C7-CLU complex, with a size range of 650-400 kDa, cannot be excluded as well. Based on the structure and binding abilities of clusterin (48), and the very high molecular weight we, however, speculate that two or more clusterin molecules may be bound to one copy of C7. On the other hand, it could well be that a “free-rider” or a bridging factor is part of the C7-CLU complex. For instance, albumin, the most abundant protein in circulation is often a constituent of protein complexes (49), thus, the very high molecular weight of the C7-CLU complex could be attributed to an association with albumin. Interestingly, low levels of the C7-CLU complex were also detected in fractions between 200 and 150 kDa, which could be due to the reconstitution of monomeric complexes. Ultimately, the observed data showcase the existence of the C7-CLU complex in circulation and lay ground for future investigation of protein-protein interactions within the complex.

Our study on the C7-CLU complex is limited by the lack of structural analysis. Electron microscopy has been extensively used to identify epitopes involved in MAC formation, providing invaluable information on the pore-forming properties of the complex (20, 50, 51). As such, a better understanding of the interaction between C7 and clusterin could be achieved by pinpointing the epitopes involved in C7-CLU complex formation. The clusterin binding site is conserved between the terminal complement proteins, as described above. Clusterin hence binds to complement domains that display a high degree of homology. The N-terminal domains of C7, C8 and C9 are highly homologous, but C7 is distinguished by the presence of 4 additional domains at the C-terminus (9, 52). Consequently, clusterin would most likely bind to C7 via the N-terminal domains. Electron microscopy will therefore be the next crucial step in assessing the exact domains that participate in the formation of the C7-CLU complex. Additionally, the use of surface plasma resonance (SPR) would provide greater insight on a potential direct interaction between highly purified C7 and clusterin, as it is a more sensitive technique that can showcase real-time binding, compared to ELISAs. Moreover, we observed that when clusterin was purified from serum, C7 was co-purified at substantially lower amounts, whereas serum-purified C7 yielded significantly higher amounts of clusterin. This was further supported by the results obtained from the C7-CLU ELISA, as the amount of the C7-CLU complex was markedly lower in serum-purified clusterin, compared to serum-purified C7. The mapping of the C7-CLU epitopes could therefore explain why the interaction between C7 and clusterin is less prominent upon capturing clusterin.

The majority of complement proteins are produced in the liver, nevertheless, the liver is not the primary site for C7 synthesis (53). Studies revealed that C7 is mainly produced in granulocytes (54), endothelial (55) and bone-marrow-derived cells (56). Thus, the mere presence or absence of C7 regulates the formation of MAC locally (57). Essentially, when C5b6 is formed, it is not regionally restricted, but circulates or diffuses to other areas. Once this complex meets locally-produced C7, the reactive lysis mechanism is initiated, causing the lysis of unsensitized cells (58). If insufficient levels of C7 are present, it acts as a limiting factor, preventing further activation, irrespective of the presence of other inhibitors (57). Another potential modulatory role for native C7 lies in its ability to bind the proenzyme plasminogen, and the active enzyme, plasmin. C7 has been reported to augment the activation of plasminogen and protect it from being degraded by α_2_-antiplasmin. Given that plasminogen was also associated with C7 incorporated in the C5b-9 complex, it is plausible that C7 could mediate plasminogen-associated inflammatory responses (59). The regulatory functions of C7 are not well understood yet, nevertheless, both C7 and clusterin have been shown to possess complement regulatory properties. This shared role could provide insight for the association between these two proteins.

Upon an immune response, a substantial amount of MAC is generated, triggering complement inhibitors like clusterin to scavenge these complexes (60). Clusterin binds to the nascent MAC before C9 molecules fully polymerize, leaving the structure in a soluble state with one to three copies of C9 (61, 62). We believe that clusterin’s ability to scavenge complement proteins plays a key role in the formation of the C7-CLU complex. Whether the formation of the complex originates in plasma or on local sites is not known, as our findings were restricted to the analysis of the C7-CLU complex in fluid-phase. Clusterin expression was reported in different types of endothelial-derived cells and has been shown to play a protective role in endothelial cell function (63–65). Furthermore, C7 acts as an anti-inflammatory decoy by trapping soluble C5b-9 on endothelial cell membranes, thereby inhibiting the excessive inflammatory effects of circulating C5b-9 (66). Given that both C7 and clusterin are expressed by endothelial cells, we cannot disregard the possibility that C7-CLU complexes form on endothelial cells. The C7-CLU complex does not appear to hinder the formation of the MAC, as we have showed that the concentration of the complex decreases upon NHS activation. Moreover, preliminary analysis does not indicate that the C7-CLU complex significantly impedes the formation of the TCC in healthy sera (**Supplementary Figure 4**). Given that the C7-CLU complex does not interfere with normal complement function, future investigations should examine whether the complex possesses a pathological role. Preliminary data collected from a subset of patients with systemic lupus erythematosus, sepsis, rheumatoid arthritis and atherosclerosis revealed that plasma concentrations of the C7-CLU complex varied between the four groups (data not shown). Future experiments will therefore concentrate on measuring C7-CLU levels in a large cohort of patient samples, using the custom C7-CLU ELISA, to assess whether the complex plays a role in complement-related diseases.

Our current findings refute the presence of an alternatively spliced C7; however, we revealed the presence of a novel complement complex formed between complement C7 and the complement inhibitor clusterin. Furthermore, we established a custom C7-CLU immunoassay that could specifically detect the complex in healthy donors, prompting further investigation of the role of the complex in homeostatic environments, in order to gain insight into the effector and regulatory functions of complement proteins in circulation, and of C7 in particular.

## Data availability statement

The original contributions presented in the study are publicly available. This data can be found here: https://www.uniprot.org/uniprotkb/P10643/entry.

## Ethics statement

The studies involving humans were approved by Ethics Committee at the Medical University of Innsbruck, Austria (ECS1166/2018, 14 November 2018). The studies were conducted in accordance with the local legislation and institutional requirements. The participants provided their written informed consent to participate in this study. The animal study was approved by Danish Animal Experiments Inspectorate (ID: 2019-15-0201-00090). The study was conducted in accordance with the local legislation and institutional requirements.

## Author contributions

MM: Conceptualization, Data curation, Investigation, Methodology, Software, Writing – original draft, Writing – review & editing. ET: Data curation, Formal Analysis, Investigation, Methodology, Resources, Supervision, Validation, Visualization, Writing – original draft, Writing – review & editing. BS: Conceptualization, Data curation, Methodology, Writing – original draft, Writing – review & editing. LK: Data curation, Methodology, Writing – original draft, Writing – review & editing. MG: Conceptualization, Investigation, Validation, Writing – original draft, Writing – review & editing. VF: Data curation, Writing – original draft. OT-Q: Data curation, Formal Analysis, Methodology, Software, Validation, Writing – review & editing. LH: Formal Analysis, Resources, Validation, Visualization, Writing – review & editing. M-OS: Conceptualization, Data curation, Methodology, Validation, Writing – review & editing. RB-O: Data curation, Methodology, Writing – review & editing. AR: Data curation, Methodology, Writing – review & editing. PG: Formal Analysis, Project administration, Resources, Validation, Writing – review & editing. DO-H: Formal Analysis, Validation, Writing – review & editing. ZP: Validation, Writing – review & editing, Formal Analysis. RW: Conceptualization, Formal Analysis, Funding acquisition, Methodology, Project administration, Resources, Supervision, Validation, Visualization, Writing – original draft, Writing – review & editing.

## References

[1] Ricklin D Hajishengallis G Yang K Lambris JD. Complement: a key system for immune surveillance and homeostasis. Nat Immunol (2010) 11(9):785–97. doi: 10.1038/ni.1923 PMC2924908 20720586

[2] Merle NS Church SE Fremeaux-Bacchi V Roumenina LT. Complement system part I - molecular mechanisms of activation and regulation. Front Immunol (2015) 6:262. doi: 10.3389/fimmu.2015.00262 26082779 PMC4451739

[3] Reis ES Mastellos DC Hajishengallis G Lambris JD. New insights into the immune functions of complement. Nat Rev Immunol (2019) 19(8):503–16. doi: 10.1038/s41577-019-0168-x PMC6667284 31048789

[4] Bubeck D. The making of a macromolecular machine: assembly of the membrane attack complex. Biochemistry (2014) 53(12):1908–15. doi: 10.1021/bi500157z 24597946

[5] Tschopp J Podack ER Müller-Eberhard HJ. Ultrastructure of the membrane attack complex of complement: detection of the tetramolecular C9-polymerizing complex C5b-8. Proc Natl Acad Sci U S A. (1982) 79(23):7474–8. doi: 10.1073/pnas.79.23.7474 PMC347362 6961424

[6] Merle NS Noe R Halbwachs-Mecarelli L Fremeaux-Bacchi V Roumenina LT. Complement system part II: role in immunity. Front Immunol (2015) 6:257. doi: 10.3389/fimmu.2015.00257 26074922 PMC4443744

[7] Morgan BP. Complement membrane attack on nucleated cells: resistance, recovery and non-lethal effects. Biochem J (1989) 264(1):1–14. doi: 10.1042/bj2640001 2690818 PMC1133540

[8] DiScipio RG Chakravarti DN Muller-Eberhard HJ Fey GH. The structure of human complement component C7 and the C5b-7 complex. J Biol Chem (1988) 263(1):549–60. doi: 10.1016/S0021-9258(19)57427-0 3335508

[9] Hobart MJ Fernie BA DiScipio RG. Structure of the human C7 gene and comparison with the C6, C8A, C8B, and C9 genes. J Immunol (1995) 154(10):5188–94. doi: 10.4049/jimmunol.154.10.5188 7730625

[10] Preissner KT Podack ER Müller-Eberhard HJ. The membrane attack complex of complement: relation of C7 to the metastable membrane binding site of the intermediate complex C5b-7. J Immunol (1985) 135(1):445–51. doi: 10.4049/jimmunol.135.1.445 3998468

[11] Barroso S Sánchez B Alvarez AJ López-Trascasa M Lanuza A Luque R. Complement component C7 deficiency in two Spanish families. Immunology (2004) 113(4):518–23. doi: 10.1111/j.1365-2567.2004.01997.x PMC1782596 15554930

[12] Fernie BA Hobart MJ. Complement C7 deficiency: seven further molecular defects and their associated marker haplotypes. Hum Genet (1998) 103(4):513–9. doi: 10.1007/s004390050859 9856499

[13] Fernie BA Orren A Sheehan G Schlesinger M Hobart MJ. Molecular bases of C7 deficiency: three different defects. J Immunol (1997) 159(2):1019–26. doi: 10.4049/jimmunol.159.2.1019 9218625

[14] O’Hara AM Fernie BA Moran AP Williams YE Connaughton JJ Orren A. C7 deficiency in an Irish family: a deletion defect which is predominant in the Irish. Clin Exp Immunol (1998) 114(3):355–61. doi: 10.1046/j.1365-2249.1998.00737.x PMC1905122 9844043

[15] Barroso S López-Trascasa M Merino D Alvarez AJ Núñez-Roldán A Sánchez B. C7 deficiency and meningococcal infection susceptibility in two spanish families. Scand J Immunol (2010) 72(1):38–43. doi: 10.1111/j.1365-3083.2010.02403.x 20591074

[16] Nishizaka H Horiuchi T Zhu ZB Fukumori Y Volanakis JE. Genetic bases of human complement C7 deficiency. J Immunol (1996) 157(9):4239–43. doi: 10.4049/jimmunol.157.9.4239 8892662

[17] Würzner R. Deficiencies of the complement MAC II gene cluster (C6, C7, C9): is subtotal C6 deficiency of particular evolutionary benefit? Clin Exp Immunol (2003) 133(2):156–9. doi: 10.1046/j.1365-2249.2003.02230.x PMC1808758 12869019

[18] Massri M Foco L Würzner R. Comprehensive update and revision of nomenclature on complement C6 and C7 variants. J Immunol (2022) 208(12):2597–612. doi: 10.4049/jimmunol.2200045 35867677

[19] Würzner R Witzel-Schlömp K Tokunaga K Fernie BA Hobart MJ Orren A. Reference typing report for complement components C6, C7 and C9 including mutations leading to deficiencies. Exp Clin Immunogenet. (1998) 15(4):268–85. doi: 10.1159/000019082 10072638

[20] Serna M Giles JL Morgan BP Bubeck D. Structural basis of complement membrane attack complex formation. Nat Commun (2016) 7:10587. doi: 10.1038/ncomms10587 26841837 PMC4743022

[21] Würzner R Hobart MJ Orren A Tokunaga K Nitze R Götze O. A novel protein polymorphism of human complement C7 detected by a monoclonal antibody. Immunogenetics (1992) 35(6):398–402. doi: 10.1007/bf00179797 1577506

[22] Köhler G Milstein C. Continuous cultures of fused cells secreting antibody of predefined specificity. Nature (1975) 256(5517):495–7. doi: 10.1038/256495a0 1172191

[23] Skjoedt MO Palarasah Y Munthe-Fog L Jie Ma Y Weiss G Skjodt K. MBL-associated serine protease-3 circulates in high serum concentrations predominantly in complex with Ficolin-3 and regulates Ficolin-3 mediated complement activation. Immunobiology (2010) 215(11):921–31. doi: 10.1016/j.imbio.2009.10.006 19939495

[24] Würzner R Fernie BA Jones AM Lachmann PJ Hobart MJ. Molecular basis of the complement C7 M/N polymorphism. A neutral amino acid substitution outside the epitope of the allospecific monoclonal antibody WU 4-15. J Immunol (1995) 154(9):4813–9. doi: 10.4049/jimmunol.154.9.4813 7722329

[25] Faserl K Chetwynd AJ Lynch I Thorn JA Lindner HH. Corona isolation method matters: capillary electrophoresis mass spectrometry based comparison of protein corona compositions following on-particle versus in-solution or in-gel digestion. Nanomaterials (Basel). (2019) 9(6):898. doi: 10.3390/nano9060898 31226785 PMC6631359

[26] Hoser SM Hoffmann A Meindl A Gamper M Fallmann J Bernhart SH. Intronic tRNAs of mitochondrial origin regulate constitutive and alternative splicing. Genome Biol (2020) 21(1):299. doi: 10.1186/s13059-020-02199-6 33292386 PMC7722341

[27] Satapathy S Dabbs RA Wilson MR. Rapid high-yield expression and purification of fully post-translationally modified recombinant clusterin and mutants. Sci Rep (2020) 10(1):14243. doi: 10.1038/s41598-020-70990-3 32859921 PMC7455699

[28] Jones SE Jomary C. Clusterin. Int J Biochem Cell Biol (2002) 34(5):427–31. doi: 10.1016/s1357-2725(01)00155-8 11906815

[29] Kapron JT Hilliard GM Lakins JN Tenniswood MP West KA Carr SA. Identification and characterization of glycosylation sites in human serum clusterin. Protein Sci (1997) 6(10):2120–33. doi: 10.1002/pro.5560061007 PMC2143570 9336835

[30] Rohne P Prochnow H Koch-Brandt C. The CLU-files: disentanglement of a mystery. Biomol Concepts. (2016) 7(1):1–15. doi: 10.1515/bmc-2015-0026 26673020

[31] Rodríguez-Rivera C Garcia MM Molina-Álvarez M González-Martín C Goicoechea C. Clusterin: Always protecting. Synthesis, function and potential issues. BioMed Pharmacother. (2021) 134:111174. doi: 10.1016/j.biopha.2020.111174 33360046

[32] Wyatt AR Yerbury JJ Berghofer P Greguric I Katsifis A Dobson CM. Clusterin facilitates *in vivo* clearance of extracellular misfolded proteins. Cell Mol Life Sci (2011) 68(23):3919–31. doi: 10.1007/s00018-011-0684-8 PMC11115182 21505792

[33] Wyatt AR Yerbury JJ Wilson MR. Structural characterization of clusterin-chaperone client protein complexes. J Biol Chem (2009) 284(33):21920–7. doi: 10.1074/jbc.M109.033688 PMC2755916 19535339

[34] Tschopp J French LE. Clusterin: modulation of complement function. Clin Exp Immunol (1994) 97 Suppl 2(Suppl 2):11–4. doi: 10.1111/j.1365-2249.1994.tb06256.x PMC1550357 8070141

[35] Tschopp J Chonn A Hertig S French LE. Clusterin, the human apolipoprotein and complement inhibitor, binds to complement C7, C8 beta, and the b domain of C9. J Immunol (1993) 151(4):2159–65. doi: 10.4049/jimmunol.151.4.2159 8345200

[36] Murphy BF Saunders JR O’Bryan MK Kirszbaum L Walker ID d’Apice AJ. SP-40,40 is an inhibitor of C5b-6-initiated haemolysis. Int Immunol (1989) 1(5):551–4. doi: 10.1093/intimm/1.5.551 2489042

[37] Choi NH Nakano Y Tobe T Mazda T Tomita M. Incorporation of SP-40,40 into the soluble membrane attack complex (SMAC, SC5b-9) of complement. Int Immunol (1990) 2(5):413–7. doi: 10.1093/intimm/2.5.413 2150757

[38] O’Bryan MK Baker HW Saunders JR Kirszbaum L Walker ID Hudson P. Human seminal clusterin (SP-40,40). Isolation and characterization. J Clin Invest. (1990) 85(5):1477–86. doi: 10.1172/jci114594 PMC296595 2185274

[39] McDonald JF Nelsestuen GL. Potent inhibition of terminal complement assembly by clusterin: characterization of its impact on C9 polymerization. Biochemistry (1997) 36(24):7464–73. doi: 10.1021/bi962895r 9200695

[40] Mollnes TE Tschopp J. A unique epitope exposed in native complement component C9 and hidden in the terminal SC5b-9 complex enables selective detection and quantification of non-activated C9. J Immunol Methods (1987) 100(1-2):215–21. doi: 10.1016/0022-1759(87)90192-x 2439601

[41] Bayly-Jones C Tran Ho BH Lau C Leung EWW D’Andrea L Lupton CJ. The neoepitope of the complement C5b-9 Membrane Attack Complex is formed by proximity of adjacent ancillary regions of C9. bioRxiv (2022) 6:42. doi: 10.1101/2022.07.06.498960 PMC9838529 36639734

[42] McCloskey MA Dankert JR Esser AF. Assembly of complement components C5b-8 and C5b-9 on lipid bilayer membranes: visualization by freeze-etch electron microscopy. Biochemistry (1989) 28(2):534–40. doi: 10.1021/bi00428a019 2713330

[43] Preissner KP Podack ER Müller-Eberhard HJ. SC5b-7, SC5b-8 and SC5b-9 complexes of complement: ultrastructure and localization of the S-protein (vitronectin) within the macromolecules. Eur J Immunol (1989) 19(1):69–75. doi: 10.1002/eji.1830190112 2465906

[44] Horiuchi T Ferrer JM Serra P Matamoros N López-Trascasa M Hashimura C. A novel nonsense mutation at Glu-631 in a Spanish family with complement component 7 deficiency. J Hum Genet (1999) 44(3):215–8. doi: 10.1007/s100380050146 10319591

[45] Grimmler M Wang Y Mund T Cilensek Z Keidel EM Waddell MB. Cdk-inhibitory activity and stability of p27Kip1 are directly regulated by oncogenic tyrosine kinases. Cell (2007) 128(2):269–80. doi: 10.1016/j.cell.2006.11.047 17254966

[46] Saibil H. Chaperone machines for protein folding, unfolding and disaggregation. Nat Rev Mol Cell Biol (2013) 14(10):630–42. doi: 10.1038/nrm3658 PMC4340576 24026055

[47] Goicoechea de Jorge E Caesar JJ Malik TH Patel M Colledge M Johnson S. Dimerization of complement factor H-related proteins modulates complement activation *in vivo* . Proc Natl Acad Sci U S A. (2013) 110(12):4685–90. doi: 10.1073/pnas.1219260110 PMC3606973 23487775

[48] Poon S Easterbrook-Smith SB Rybchyn MS Carver JA Wilson MR. Clusterin is an ATP-independent chaperone with very broad substrate specificity that stabilizes stressed proteins in a folding-competent state. Biochemistry (2000) 39(51):15953–60. doi: 10.1021/bi002189x 11123922

[49] Marini I Moschini R Del Corso A Mura U. Chaperone-like features of bovine serum albumin: a comparison with alpha-crystallin. Cell Mol Life Sci (2005) 62(24):3092–9. doi: 10.1007/s00018-005-5397-4 PMC11138380 16314918

[50] Podack ER. Molecular composition of the tubular structure of the membrane attack complex of complement. J Biol Chem (1984) 259(13):8641–7. doi: 10.1016/S0021-9258(17)39778-8 6736043

[51] Hadders MA Bubeck D Roversi P Hakobyan S Forneris F Morgan BP. Assembly and regulation of the membrane attack complex based on structures of C5b6 and sC5b9. Cell Rep (2012) 1(3):200–7. doi: 10.1016/j.celrep.2012.02.003 PMC3314296 22832194

[52] Tegla CA Cudrici C Patel S Trippe R 3rd Rus V Niculescu F. Membrane attack by complement: the assembly and biology of terminal complement complexes. Immunol Res (2011) 51(1):45–60. doi: 10.1007/s12026-011-8239-5 21850539 PMC3732183

[53] Würzner R Joysey VC Lachmann PJ. Complement component C7. Assessment of *in vivo* synthesis after liver transplantation reveals that hepatocytes do not synthesize the majority of human C7. J Immunol (1994) 152(9):4624–9. doi: 10.4049/jimmunol.152.9.4624 8157976

[54] Høgåsen AK Würzner R Abrahamsen TG Dierich MP. Human polymorphonuclear leukocytes store large amounts of terminal complement components C7 and C6, which may be released on stimulation. J Immunol (1995) 154(9):4734–40. doi: 10.4049/jimmunol.154.9.4734 7722325

[55] Langeggen H Pausa M Johnson E Casarsa C Tedesco F. The endothelium is an extrahepatic site of synthesis of the seventh component of the complement system. Clin Exp Immunol (2000) 121(1):69–76. doi: 10.1046/j.1365-2249.2000.01238.x 10886241 PMC1905676

[56] Naughton MA Walport MJ Würzner R Carter MJ Alexander GJ Goldman JM. Organ-specific contribution to circulating C7 levels by the bone marrow and liver in humans. Eur J Immunol (1996) 26(9):2108–12. doi: 10.1002/eji.1830260922 8814254

[57] Würzner R. Modulation of complement membrane attack by local C7 synthesis. Clin Exp Immunol (2000) 121(1):8–10. doi: 10.1046/j.1365-2249.2000.01263.x 10886232 PMC1905667

[58] Thompson RA Lachmann PJ. Reactive lysis: the complement-mediated lysis of unsensitized cells. I. The characterization of the indicator factor and its identification as C7. J Exp Med (1970) 131(4):629–41. doi: 10.1084/jem.131.4.629 PMC2138783 4193934

[59] Reinartz J Hänsch GM Kramer MD. Complement component C7 is a plasminogen-binding protein. J Immunol (1995) 154(2):844–50. doi: 10.4049/jimmunol.154.2.844 7814888

[60] Barnum SR Bubeck D Schein TN. Soluble membrane attack complex: biochemistry and immunobiology. Front Immunol (2020) 11:585108. doi: 10.3389/fimmu.2020.585108 33240274 PMC7683570

[61] Menny A Lukassen MV Couves EC Franc V Heck AJR Bubeck D. Structural basis of soluble membrane attack complex packaging for clearance. Nat Commun (2021) 12(1):6086. doi: 10.1038/s41467-021-26366-w 34667172 PMC8526713

[62] Murphy BF Kirszbaum L Walker ID d’Apice AJ. SP-40,40, a newly identified normal human serum protein found in the SC5b-9 complex of complement and in the immune deposits in glomerulonephritis. J Clin Invest. (1988) 81(6):1858–64. doi: 10.1172/jci113531 PMC442636 2454950

[63] Franco DA Truran S Burciu C Gutterman DD Maltagliati A Weissig V. Protective role of clusterin in preserving endothelial function in AL amyloidosis. Atherosclerosis (2012) 225(1):220–3. doi: 10.1016/j.atherosclerosis.2012.08.028 PMC3478430 22981431

[64] Kim M Lee J Park TJ Kang HY. Paracrine crosstalk between endothelial cells and melanocytes through clusterin to inhibit pigmentation. Exp Dermatol (2018) 27(1):98–100. doi: 10.1111/exd.13443 28887822

[65] Mishima K Inoue H Nishiyama T Mabuchi Y Amano Y Ide F. Transplantation of side population cells restores the function of damaged exocrine glands through clusterin. Stem Cells (2012) 30(9):1925–37. doi: 10.1002/stem.1173 22782911

[66] Bossi F Rizzi L Bulla R Debeus A Tripodo C Picotti P. C7 is expressed on endothelial cells as a trap for the assembling terminal complement complex and may exert anti-inflammatory function. Blood (2009) 113(15):3640–8. doi: 10.1182/blood-2008-03-146472 19179470

