# Supplementary material for: Complement C7 and clusterin form a complex in circulation
Source: Front Immunol. 2024 Jan 25;15:1330095. doi: 10.3389/fimmu.2024.1330095 (PMC10850381; doi:10.3389/fimmu.2024.1330095)
Supplement: Supplementary file 1 [file DataSheet_1.docx]

Supplementary Material

**Supplementary Figure 1.** **Characterization of M7-HB2H as a native-restricted C7 mAb.** The concentration of C7 detected in NHS, using the M7-HB2H mAb, decreases upon activation of NHS to below 5%, indicating that the M7-HB2H mAb is native-restricted. Some error bars cannot be shown because the SD is too small.


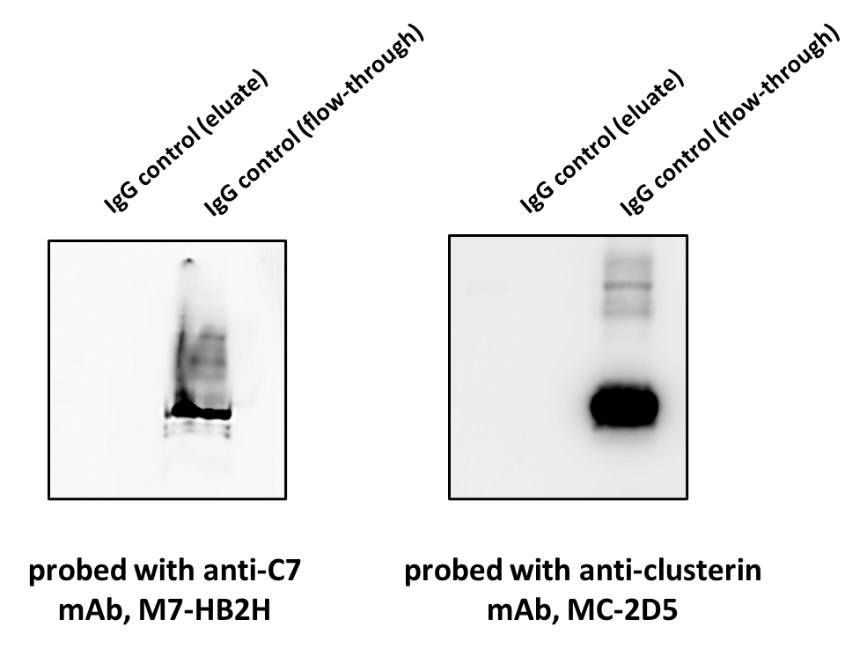


Supplementary Figure 2. No C7 or clusterin detected in eluates acquired using IgG isotype control. Eluates and flow-throughs acquired from resin-based column coupled with an IgG isotype control were separated by SDS-PAGE under non-reducing conditions and immunoblots were probed with the anti-C7 mAb, M7-HB2H, or the anti-clusterin mAb, MC-2D5. mAb, monoclonal antibody.


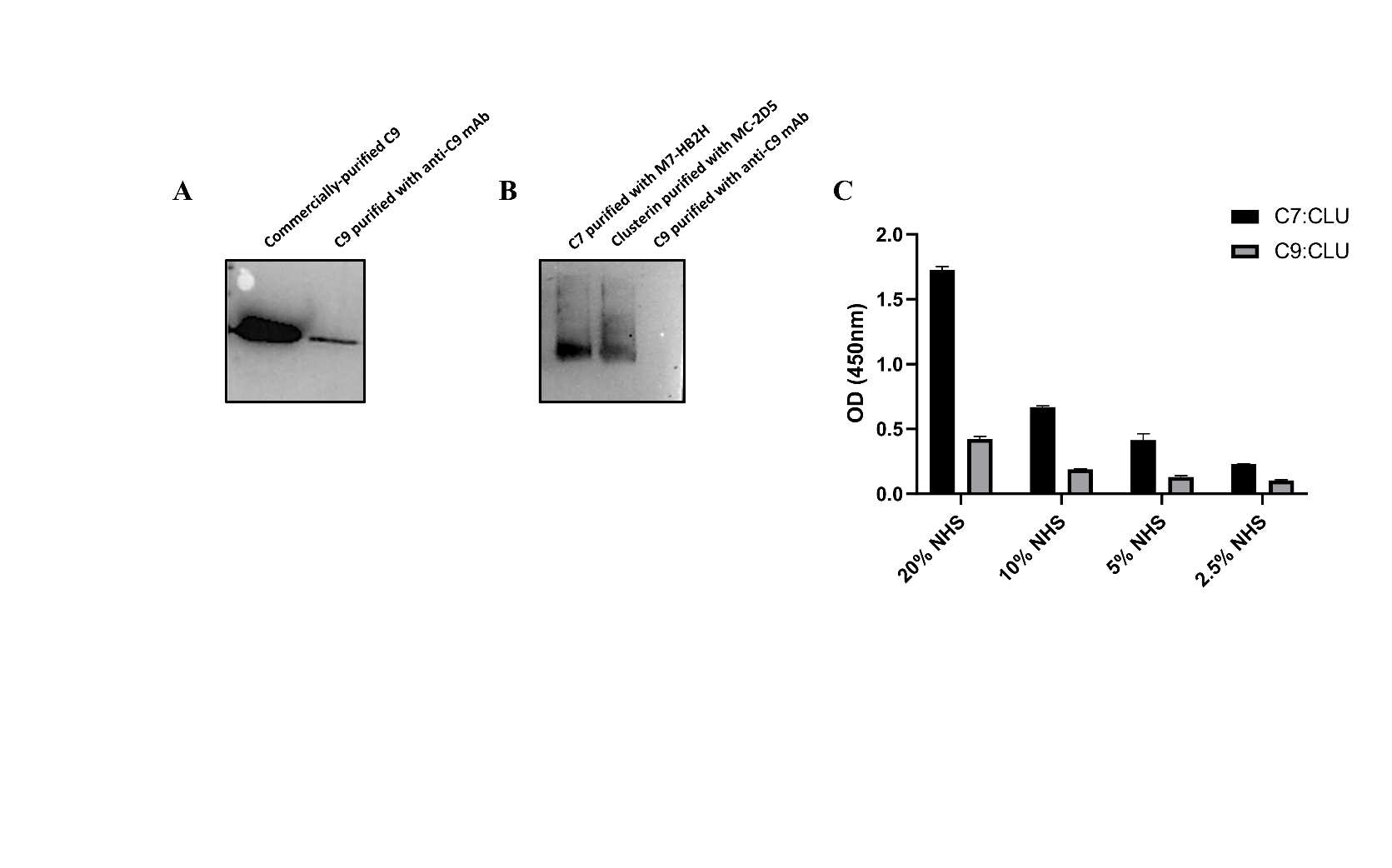


Supplementary Figure 3. Serum-purified C9 does not appear to associate with clusterin. (A) Commercially-purified C9 and C9 purified from serum (NHS-SU) with an anti-C9 mAb were separated in an SDS-PAGE gel under non-reducing conditions and probed with an anti-C9 mAb. (B) Serum-purified C7, clusterin and C9 were separated on an SDS-PAGE gel under non-reducing condition and probed with the anti-clusterin mAb, MC-2D5. (C) The interaction between C7 and clusterin and C9 and clusterin was measured in 20%-2.5% NHS by ELISA using the following coat/detection combinations: anti-C7 mAb/anti-clusterin mAb (C7/CLU) or anti-C9 mAb/anti-clusterin mAb (C9/CLU). NHS, normal human serum.


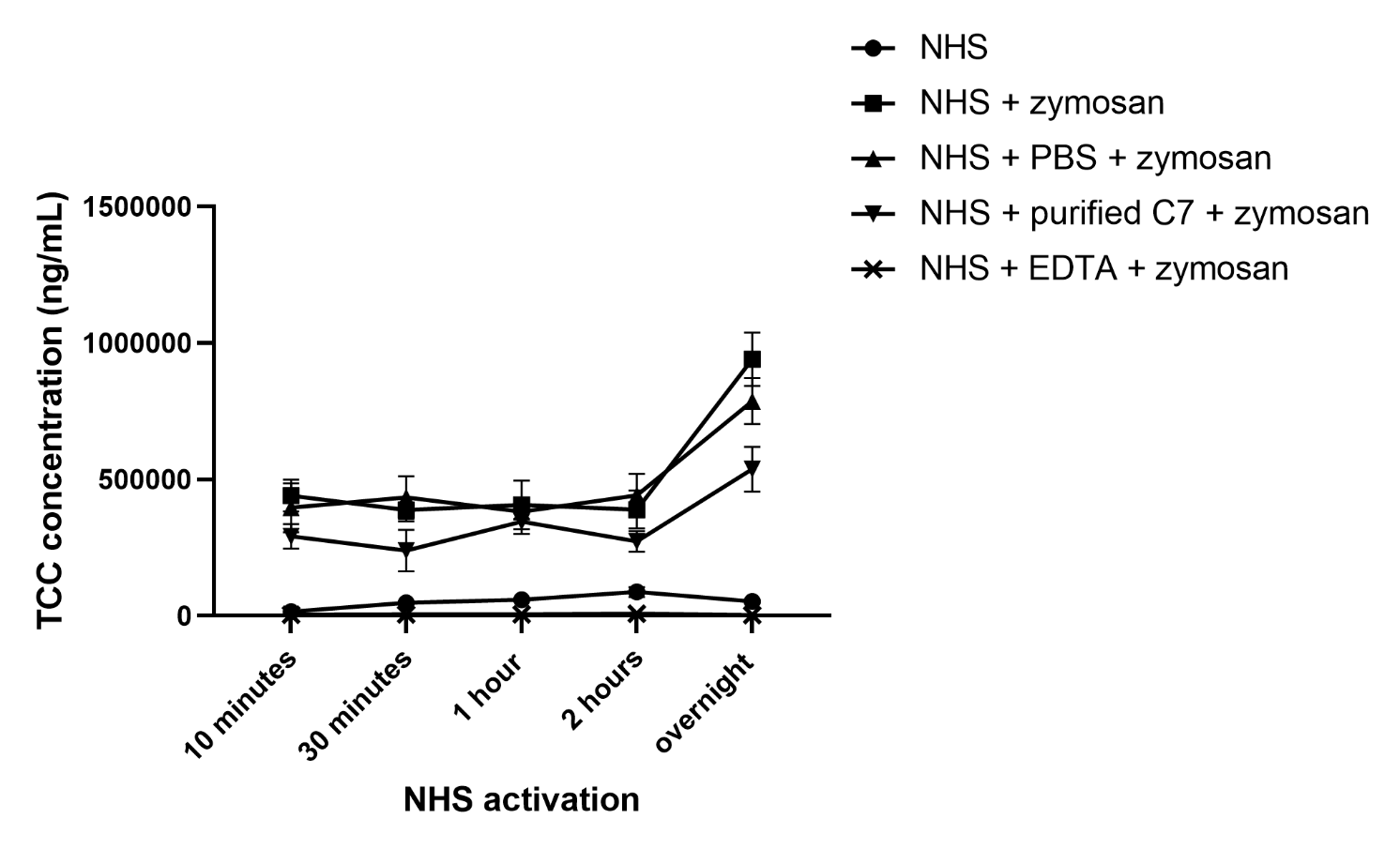


Supplementary Figure 4. The C7-CLU complex does not affect the formation of the TCC. TCC levels were measured in NHS incubated with indicated reagents for 30 minutes at RT and subsequently activated with zymosan at 37^o^C for 10 minutes, 30 minutes, 1 hour, 2 hours or overnight. EDTA, ethylenediaminetetraacetic acid; NHS, normal human serum; ns, not significant; PBS, phosphate buffered saline; RT, room temperature; TCC, terminal complement complex.

**Supplementary Table 1. List of the 20 most abundant proteins detected in serum-purified C7.**

| Protein FDR Confidence | Accession | Protein | Coverage [%] | # Peptides | # AAs | MW [kDa] | Abundance |
| --- | --- | --- | --- | --- | --- | --- | --- |
| High | P10643 | Complement C7 | 70 | 78 | 843 | 93.5 | 1.01E+11 |
| High | P10909 | Clusterin | 60 | 40 | 449 | 52.5 | 7.38E+10 |
| High | P02768 | Albumin | 91 | 88 | 609 | 69.3 | 4.61E+10 |
| High | P01871 | Immunoglobulin heavy constant mu | 68 | 37 | 453 | 49.4 | 2.36E+10 |
| High | P01024 | Complement C3 | 73 | 139 | 1663 | 187 | 2.16E+10 |
| High | P02765 | Alpha-2-HS-glycoprotein | 48 | 19 | 367 | 39.3 | 1.60E+10 |
| High | P01876 | Immunoglobulin heavy constant alpha | 67 | 18 | 353 | 37.6 | 1.25E+10 |
| High | P00751 | Complement factor B | 58 | 46 | 764 | 85.5 | 8.84E+09 |
| High | P02743 | Serum amyloid  P-component | 35 | 13 | 223 | 25.4 | 8.43E+09 |
| High | P00747 | Plasminogen | 73 | 65 | 810 | 90.5 | 7.29E+09 |

Protein composition of C7 purified from NHS-BioIVT with the anti-C7 mAb, M7-HB2H, was analyzed by mass spectrometry. The 10 most abundant proteins in the purified C7 sample are shown. The abundance indicates the sum of all peptide ions for the detected proteins. FDR: false discovery rate.
